# Emissions of methane from offshore oil and gas platforms in Southeast Asia

**DOI:** 10.1038/srep06503

**Published:** 2014-09-30

**Authors:** Hideki Nara, Hiroshi Tanimoto, Yasunori Tohjima, Hitoshi Mukai, Yukihiro Nojiri, Toshinobu Machida

**Affiliations:** 1 Center for Global Environmental Research, National Institute for Environmental Studies, 16-2 Onogawa, Tsukuba, Ibaraki 305-8506, Japan

## Abstract

Methane is a substantial contributor to climate change. It also contributes to maintaining the background levels of tropospheric ozone. Among a variety of CH_4_ sources, current estimates suggest that CH_4_ emissions from oil and gas processes account for approximately 20% of worldwide anthropogenic emissions. Here, we report on observational evidence of CH_4_ emissions from offshore oil and gas platforms in Southeast Asia, detected by a highly time-resolved spectroscopic monitoring technique deployed onboard cargo ships of opportunity. We often encountered CH_4_ plumes originating from operational flaring/venting and fugitive emissions off the coast of the Malay Peninsula and Borneo. Using night-light imagery from satellites, we discovered more offshore platforms in this region than are accounted for in the emission inventory. Our results demonstrate that current knowledge regarding CH_4_ emissions from offshore platforms in Southeast Asia has considerable uncertainty and therefore, emission inventories used for modeling and assessment need to be re-examined.

Atmospheric CH_4_ is an important component of short-lived climate pollutants that contribute both directly and indirectly to radiative forcing. It is also known that CH_4_ contributes to maintaining the background levels of tropospheric ozone^1^. CH_4_ is emitted from a variety of natural (e.g., wetlands, oceans, termites, and clathrates) and anthropogenic (e.g., fossil-fuel exploitation, ruminant animals, rice cultivation, waste management, and biomass burning) sources. Because of the shorter atmospheric lifetime (about nine years^2^) of CH_4_ than CO_2_, a reduction of anthropogenic emissions of CH_4_ would be an effective means of abating global warming in the near future^3^,^4^. However, to establish strategies for the mitigation of global warming, a quantitative understanding of the global CH_4_ budget is required.

Atmospheric abundance of CH_4_ has been increasing from pre-industrial levels of about 700 nmol mol^−1^ (hereafter referred to as ppb) with large year-to-year fluctuations in its growth rate^5^. Among the many studies that have investigated the distribution and temporal variation of CH_4_, several have reported conflicting results^6^,^7^,^8^,^9^. Some recent studies have suggested the existence of previously unrecognized sources of CH_4_. For example, satellite observations have been combined with inverse modeling techniques using CH_4_ retrievals from the Scanning Imaging Absorption Spectrometer for Atmospheric Chartography (SCIAMACHY) to provide a global distribution map of CH_4_ that suggests there are many emission hot spots in areas where surface observations are scarce^10^. The first airborne in situ measurements of CH_4_ over the Amazon region during the BARCA (Balanço Atmosphérico Regional de Carbono na Amazônia) campaign revealed strong CH_4_ emissions from the Amazonian wetlands^11^. Regular aircraft observations from the CARIBIC (Civil Aircraft for the Regular Investigation of the atmosphere Based on an Instrument Container) program suggest strong biogenic emissions from India that cannot be attributed solely to rice cultivation^12^. These studies show that our current understanding regarding the sources of CH_4_ emissions is inadequate and that greater effort is needed to obtain better knowledge both of the strength of CH_4_ emissions and of the distribution of the sources. For this purpose, a more systematic approach is required regarding the acquisition of CH_4_ observations in areas where observational data are sporadic or sparse.

Since 1992, the National Institute for Environmental Studies (NIES) has conducted a voluntary observing ships (VOS) program of long-term atmospheric monitoring of climatically important trace gases over the Pacific Ocean^13^,^14^. In the Southeast Asian region, atmospheric monitoring as part of the NIES-VOS program commenced in September 2007. Although flask sampling was initially used for CH_4_ monitoring, since 2009, the program has been augmented by the use of continuous measurements that capture the highly variable features of CH_4_ in the regionally polluted air in Southeast Asia. In this paper, we present the first results of the high-resolution continuous onboard measurements of CH_4_ in the marine boundary layer (MBL) in the Southeast Asian region between September 2009 and April 2012. We focus on the CH_4_ distribution in the northern equatorial region, where strong CH_4_ peaks were observed off the east coast of the Malay Peninsula and the northwest coast of Borneo, and we examine the emission sources responsible for these observed CH_4_ peaks.

## Results

**Figure 1:**
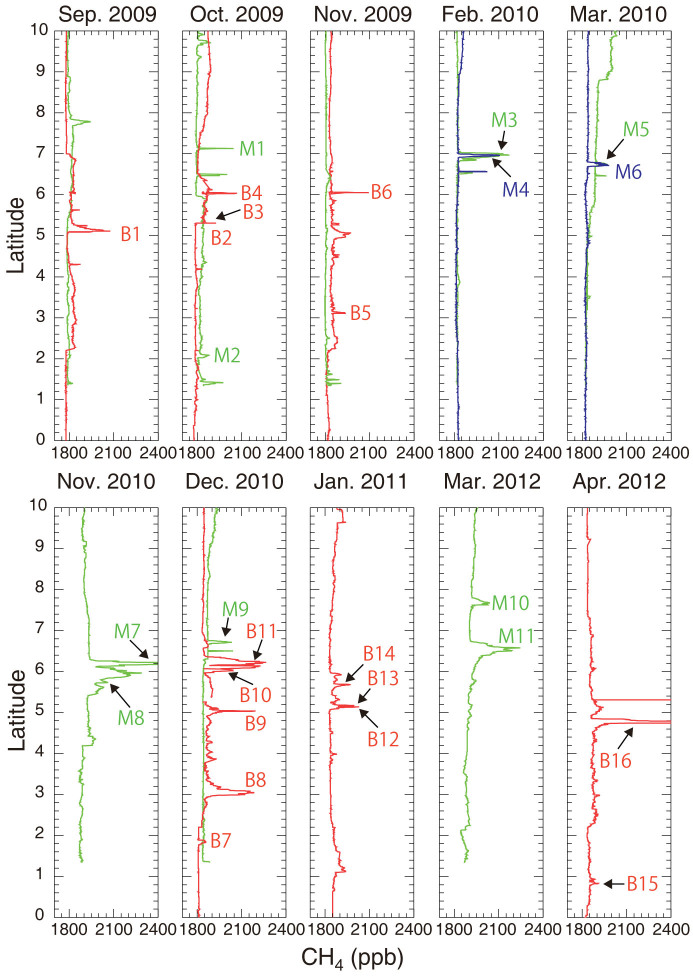
Latitudinal distribution of 1-min temporally averaged CH_4_ mole fractions between latitude 10°N and the equator observed during eight voyages along the Southeast Asian shipping routes between September 2009 and April 2012. Mole fractions of CH_4_ are color coded according to the shipping routes: green for the southbound Asia route, blue for northbound Asia route, and red for northbound Borneo route. Peak numbers are allocated for only the Malay and Borneo peaks (preceded by M or B) that showed substantial CH_4_ increase (>50 ppb) and positive correlation between CH_4_ and CO_2_ (*R* > 0.4, p-value < 0.05) and that had peak durations of more than 10 min.

Although shipping operations were disrupted temporarily by the global financial crisis and the 2011 Great East Japan Earthquake, we were able to undertake observations during eight voyages between September 2009 and April 2012. Many CH_4_ peaks were observed in the northern equatorial region along Southeast Asian routes (Figure 1); see Methods section for further details. The locations at which these peaks occurred were concentrated in two areas: off the east coast of the Malay Peninsula (39 peaks) and off the northwest coast of Borneo (55 peaks); they are referred to hereafter as the Malay and Borneo peaks, respectively. The Malay peaks were observed largely between latitudes 8°N and 5.5°N along both the northbound and southbound routes, while the Borneo peaks were observed between latitudes 6.5°N and 4.5°N along the northbound Borneo route. Although the durations of all observed CH_4_ peaks were short, between several minutes to one hour, the increases of the mole fraction of CH_4_ were considerable, i.e., up to about 1100 ppb above the baseline levels for the Southeast Asian region. Concurrent with the CH_4_ peaks, we observed simultaneous CO_2_ peaks, and the positive correlation between these CO_2_ and CH_4_ mole fractions suggested a common local, non-biogenic emission source for these gases.

**Figure 2:**
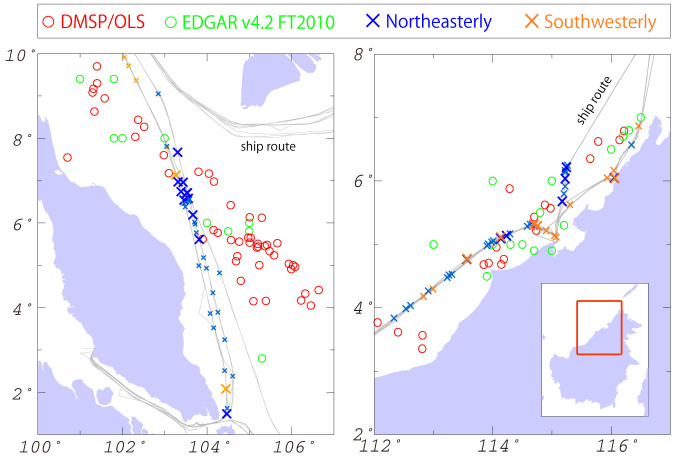
Distribution of CH_4_ peaks observed during this study and offshore platforms off the east coast of the Malay Peninsula (left) and the northwest coast of Borneo (right). Crosses are the locations where CH_4_ peaks were observed and their colors explain wind patterns when each CH_4_ peak was observed (blue: northeasterly wind; orange: southwesterly wind). Numbered peaks in Figure 1 are emphasized as large crosses, while other marginal peaks are shown by small crosses. Open red and green circles are the locations of offshore platforms in 2010, identified based on DMSP/OLS data and as reported in the EDGAR v.4.2 FT2010 database, respectively. Gray solid lines mark the routes of the VOS ships. The maps used in this figure were generated by Generic Mapping Tools (https://www.soest.hawaii.edu/gmt/).

To identify sources of CH_4_ emission, we examined satellite-observed night-light data from the US Air Force Defense Meteorological Satellite Project Operational Linescan System (DMSP/OLS), provided by the US National Oceanic and Atmosphere Administration^15^. Using the nighttime lights data “avg lights x pct”, which are annual composite images of noise-filtered nighttime lights data used to infer gas-flaring volumes^16^, we identified the locations of offshore platforms within the study area. The distribution of these identified offshore platforms remained largely unchanged throughout our study period. Most of these platforms were either off the east coast of the Malay Peninsula or off the northwest coast of Borneo and were near the locations of the CH_4_ peaks along the Southeast Asian trade routes (Figure 2). Generally, CH_4_ is a dominant component of emissions from offshore oil platforms, released as a result of gas flaring and venting, equipment leaks, and evaporation losses, with concomitant emissions of CO_2_ mainly due to gas flaring^16^,^17^. These results suggest that the observed CH_4_ peaks represent emissions from offshore production platforms.

The CH_4_ emissions from offshore platforms are reported in the anthropogenic trace gas emission inventory database EDGAR (Emission Database for Global Atmospheric Research) v.4.2 FT2010^18^. We compared the distribution of offshore platforms identified in this study to that reported in EDGAR. For the comparison, we used the annual composite image for 2010 from the DMSP/OLS data and annual CH_4_ emission data for 2010 from EDGAR. This revealed considerable discrepancy in the distribution of offshore platforms, especially off the east coast of the Malay Peninsula, which indicates that the current emission inventories of offshore platforms in Southeast Asia still include considerable uncertainties regarding CH_4_ and other co-emitted gas components.

Most of our observations were performed during the boreal fall and winter season when strong northeasterly winds associated with the East Asian monsoon prevail off the east coast of the Malay Peninsula; westerly winds passing over the Malay Peninsula from the Indian Ocean prevailed only during September and October of 2009 during our observations. In contrast, there was no prevailing wind direction off the northwest coastal region of Borneo during our study period. Thus, the Malay peaks observed during the northeasterly wind season should represent emissions from offshore platforms windward of the peak locations, whereas the Borneo peaks represent emissions from both offshore platforms and onshore coastal sources. To characterize the offshore platform emissions, we examined the emissions measured during the CH_4_ peaks based on the CH_4_–CO_2_ enhancement ratio (ΔCH_4_/ΔCO_2_), which is the linear slope of the correlation of the mole fractions of CH_4_ and CO_2_. The observed enhancement ratio can often be used to identify the emission sources because it can be approximated to the emission ratio when observations are performed near the emission sources. For example, past observations at remote sites during wintertime have shown the ΔCH_4_/ΔCO_2_ ratios are typically less than about 20 ppb/ppm (ppm is defined as μmol mol^−1^) in air masses polluted principally by anthropogenic combustion-related emissions in urban and industrialized areas^19^,^20^,^21^,^22^. The emission factors of CO_2_ and CH_4_ from biomass-burning sources were determined the typical CH_4_/CO_2_ ratios to be less than 20 ppb/ppm^23^. These results provide diagnostic criteria for estimating the contributions from these anthropogenic emissions on land.

**Figure 3:**
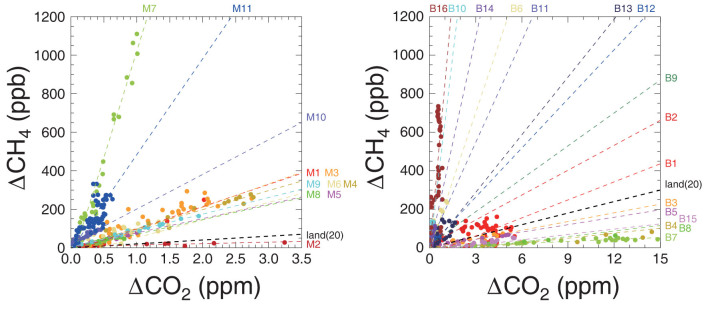
Scatter plots of CH_4_ versus CO_2_ mole fractions during observed CH_4_ peaks. Numbering of peaks is as described in Figure 1. The left and right panels are for the Malay and Borneo peaks, respectively. Dashed lines indicate regression lines for individual peaks determined by reduced major-axis regression. The black dashed line labeled “land(20)” indicates the upper limit of the CH_4_–CO_2_ emission ratio for onshore anthropogenic emissions.

For further analysis of the ΔCH_4_/ΔCO_2_ ratios observed here, we selected 11 distinct Malay peaks and 16 Borneo peaks that showed substantial CH_4_ increases (>50 ppb) for more than 10 min and significant positive correlations between CH_4_ and CO_2_ (*R* > 0.4, p-value < 0.05). The ΔCH_4_/ΔCO_2_ ratios during these peaks, calculated by reduced major-axis regression^24^, ranged from 8 to 1108 ppb/ppm and from 3 to 880 ppb/ppm for the Malay and Borneo peaks, respectively (Figure 3). The M1, M3, M4, M5, M6, M8, and M9 peaks show similar ΔCO_2_-vs.-ΔCH_4_ correlative behavior, suggesting that they originated from the same emission process at the offshore platforms. We examined the approximate flaring efficiency (i.e., ΔCO_2_/(ΔCO_2_ + ΔCH_4_) in %) using the ΔCO_2_-vs.-(ΔCO_2_ + ΔCH_4_) regression for these peaks. The mean ΔCH_4_/ΔCO_2_ ratio for these peaks is 94 ppb/ppm, corresponding to a flaring efficiency of 92%. As gas-flaring efficiencies for industrial flares are usually greater than 90%, it is considered that these plumes originated principally from flaring with the contribution from fugitive emissions (inclusive of venting), if any, being relatively small. The M7, M10, and M11 peaks show higher ΔCH_4_/ΔCO_2_ ratios than those of the gas-flaring plumes, suggesting greater contribution from fugitive emissions. These results indicate that the observed ΔCH_4_/ΔCO_2_ ratios can vary widely, depending on the contributions from fugitive emissions. In contrast, the contributions from flaring and fugitive emissions to the M2 peak, associated with the ΔCH_4_/ΔCO_2_ ratio of 8 ppb/ppm, appear negligible. Similarly, the 16 peaks observed in the Borneo area with ΔCH_4_/ΔCO_2_ ratios higher than 20 ppb/ppm were explained by the mixing of flaring and fugitive emissions. Consequently, we chose those peaks with ΔCH_4_/ΔCO_2_ ratios higher than 20 ppb/ppm for further analysis.

## Discussion

We used these observed CH_4_ peaks to estimate the CH_4_ emission rates based on a mass balance approach^25^,^26^,^27^. Assuming that the CH_4_ plume was formed steadily during the observation and that the CH_4_ mixing ratio was vertically well mixed in the MBL, the CH_4_ emission rate *q_CH4_* can be expressed by: 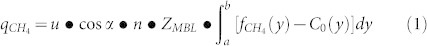
In equation (1), *u* is the mean horizontal wind speed along the plume axis, *α* is the angle between the ship transect and the perpendicular to the plume axis, *Z_MBL_* is the depth of the MBL, *n* is the average molar density of air within the MBL, *y* is the distance from the plume axis, *f*_CH4_(*y*) is the observed CH_4_ mole fraction at *y*, and *C_0_(y)* is the background CH_4_ mole fraction at *y*. The start and end points of the integration interval for the individual CH_4_ peaks, *a* and *b* in equation (1), are determined manually by visual inspection, and the values of *C_0_(y)* are determined practically by linear interpolation between the CH_4_ mole fractions at points *a* and *b*. As no meteorological observations were performed onboard, the mean wind speeds and directions and the depths of the MBL were estimated based on the CGER/METEX three-dimensional kinematic trajectory model^28^. This trajectory model was driven by six-hourly meteorological input data from the NCEP/NCAR reanalysis, which has a spatial resolution of 2.5° × 2.5°. The model calculation was initiated at an altitude of 250 m above sea level at the locations of the CH_4_ peaks. For every plume calculation, we adopted the molar density of air (*n*) of 1.2 kg m^−3^, which was the average value of *n* at 0 and 0.5 km^29^. We applied the mass balance approach to the appropriate 14 peaks that the ship transited straight across the CH_4_ peak. Geographical relationships between the observed CH_4_ peaks and the offshore platforms were well explained by the trajectory model. The resultant CH_4_ emission rates for the eight Malay platform peaks: M1, M3, M4, M5, M6, M7, M8, and M9 are within the range of 3.9–426.7 g s^−1^ with a median (mean) of 99.2 (124.7) g s^−1^. The rates for the six Borneo platform peaks: B1, B6, B11, B12, B13, and B14 are within the range of 1.8–46.0 g s^−1^ with a median (mean) of 14.7 (16.9) g s^−1^, as summarized in Table 1, together with the values of the parameters used in the calculations. These estimates are comparable with the recent preliminary estimates using the Visible Infrared Imaging Radiometer Suite on board the Suomi National Polar-orbiting Partnership satellite, provided by the National Geophysical Data Center of the National Oceanic and Atmospheric Administration^30^. As conservative error estimates, we evaluated uncertainty ranges for the emission rates by assuming the relative uncertainty of ±50% for the two dominant factors (u × cosθ and Z_MBL_), and ±0.05 kg m^−3^ for the molar density of air (1.2 kg m^−3^). The calculated uncertainty ranges (from lower to upper limits), also listed in Table 1, suggest substantial uncertainty in this approach.

The median value of all the CH_4_ emission rates is 29.2 g s^−1^. Using the DMSP satellite, we identified 112 offshore platforms in the Southeast Asian region (defined as: 15°N–10°S, 90°–140°E); thus, the resultant regional total emission rate is calculated as 3.3 kg s^−1^. The total regional annual emission of CH_4_ from offshore platforms in the Southeast Asian region is estimated to be about 0.1 Tg y^−1^, associated with an uncertainty range of 0.02–0.32 Tg y^−1^ (the median values of the lower and upper limits). EDGAR reports that annual CH_4_ emissions from oil and gas production in 12 Southeast Asian countries (Brunei, Cambodia, Lao, Myanmar, Malaysia, the Philippines, Singapore, Thailand, Timor, Vietnam, Indonesia, and Papua New Guinea) were about 3.7 Tg y^−1^ for 2010, which corresponds to about 1% of the 335 Tg y^−1^ global anthropogenic CH_4_ emissions^18^. Offshore CH_4_ emissions account for about 8% (0.29 Tg y^−1^) of the total emissions from oil and gas production in the Southeast Asian region. Despite the large uncertainty inherent in the mass balance approach, our estimate displays relatively good agreement with that by EDGAR. However, we note substantial differences in the locations of the offshore platforms between the EDGAR inventory and those determined by DMSP satellite observation. The distributions of point sources of CH_4_ are an important uncertainty in the existing inventories. The relative contributions of the offshore CH_4_ emissions to the regional CH_4_ emissions in Southeast Asia are estimated to be about 3% for the oil and gas production sector (both offshore and onshore) (3.7 Tg y^−1^, as estimated by EDGAR), and about 0.2% for the anthropogenic sources (63 Tg y^−1^, as estimated by EDGAR).

The global CH_4_ emissions in 2011 are estimated to be 556 ± 56 Tg y^−1^, with contributions from natural and anthropogenic emissions being comparable^31^. Natural emissions from wetlands are the single most dominant contributor to the total CH_4_ emissions, with the annual emissions being approximately 200 Tg y^−1^. The middle-class sources with the annual emissions in the range of 10–100 Tg y^−1^ include fossil fuels, ruminants, landfills/waste, geological sources, freshwater, rice paddies, burning of biomass/biofuels, wild animals, and termites. The emissions from other sources are minor. The emissions (and range) from hydrates, wildfires, and permafrost are estimated to be 6 (2–9), 3 (1–5), and 1 (0–1) Tg y^−1^, respectively.

Globally, there are a number of offshore fields for oil and gas production. In addition to Southeast Asia, the North Sea, Persian Gulf, Gulf of Guinea, and Gulf of Mexico are known to be active in oil and gas production. As noted above, our estimate and EDGAR are in relatively good agreement for offshore CH_4_ emissions in Southeast Asia. A simple global estimate based on EDGAR implies that CH_4_ emissions from worldwide offshore oil and gas platforms are 1–2 Tg y^−1^, suggesting that the emissions from offshore sources may be comparable to those from minor natural sources such as wildfires and permafrost.

To our knowledge, this work marks the first top-down constraint on CH_4_ emissions from oil and gas platforms in Southeast Asia. On the other hand, we also realize the considerable uncertainty in our estimates, which derive from a combination of features inherent in the mass balance approach and the lack of samples of CH_4_ plumes from offshore platforms, due to the sporadic occurrence of gas flaring and fugitive emissions at oil and gas platforms. Hence, our top-down estimates of CH_4_ emissions from offshore platforms located in the Southeast Asian region need to be tested and improved. For example, if fugitive plumes were undersampled in our observations, the estimated CH_4_ emissions would be much greater, possibly even by one order of magnitude. To better assess the regional total emissions of CH_4_ from offshore platforms and thereby improve the current emissions inventory, further top-down constraints by integrated ship, aircraft, and satellite observations are needed. In particular, the detection of fugitive plumes would be useful to reduce uncertainties in estimating the emissions. Current estimates of CH_4_ emissions from oil and gas processes were approximately 20% of worldwide anthropogenic emissions in 2010, and they are expected to increase by nearly 35% between 2010 and 2020^32^. The feedback gained from plume observations can help in the reduction of fugitive emissions in Southeast Asian countries and thus, contribute to the mitigation of global warming.

## Methods

**Figure 4:**
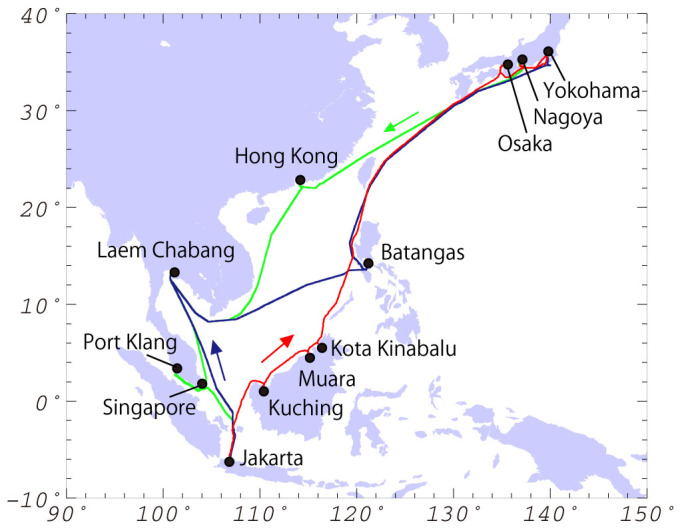
The VOS shipping routes in Southeast Asia. Green and blue lines show the southbound (Japan–Indonesia) and northbound (Indonesia–Japan) Asia routes respectively; a red line shows the northbound Borneo route (Indonesia–Japan). Regular berthing ports are shown as solid black circles. The maps used in this figure were generated by Generic Mapping Tools (https://www.soest.hawaii.edu/gmt/).

We used two commercial cargo vessels in the VOS program in Southeast Asia: the M/V *Fujitrans World* (owned by the Kagoshima Senpaku Kaisya, Ltd., Japan) was the primary vessel with backup provided by the M/V *Trans Future 1* (owned by the Toyofuji Shipping Co. Ltd., Japan). These ships regularly sail the trade routes between Japan and Southeast Asia, berthing at Osaka, Yokohama, and Nagoya (Japan); Hong Kong (China); Laem Chabang (Thailand); Singapore; Port Klang, Kuching and Kota Kinabalu (Malaysia); Jakarta (Indonesia); and Muara (Brunei) at four-week intervals (Figure 4). Two northbound routes are used from Jakarta to Japan: one via Thailand and the Philippines (the northbound Asia route) and the other via Borneo (the northbound Borneo route). Only one southbound route is used from Japan to Indonesia.

Onboard each VOS ship, continuous measurements of CO_2_, CO, and O_3_ were performed using a non-dispersive infrared analyzer (NDIR), an NDIR with gas filter correlation, and an ultraviolet absorption analyzer, respectively. The continuous CO_2_ data processed for public use are available at our webpage (http://soop.jp/). Flask samples were also collected for laboratory analysis of CO_2_, CO, CH_4_, N_2_O, SF_6_, H_2_, O_2_/N_2_, and CO_2_ isotopologues (^13^CO_2_, ^12^C^18^O^16^O). A detailed description of the atmospheric observation system is provided elsewhere^13^.

In September 2009, continuous measurements of atmospheric CO_2_ and CH_4_ were commenced on VOS ships along both of the Southeast Asian routes using wavelength-scanned cavity ring-down spectroscopy (WS-CRDS) instruments (Picarro Inc., Santa Clara, CA, USA, models EnviroSense 3000i and G1301). Air sample was collected from the air intake set at the top deck of the ship (approximately 50 m above sea level) using a diaphragm pump placed in the observation room. The sampled air was dried before analysis to minimize biases due to dilution and pressure-broadening effects of water vapor on the WS-CRDS measurements. The sampled air was dehumidified by passing it through a sample-drying unit consisting of an electric cooler kept at +1°C and a Nafion Perma Pure dryer (Perma Pure LLC, Toms River, NJ, USA). The design and performance of the unit was very similar to that used for the CO measurements^13^. The sampled air was dried to less than ~0.3% water (as measured by the WS-CRDS) before the mole fractions of CO_2_ and CH_4_ were determined based on the water vapor content according to an instrument-specific water vapor collection function^33^. To our experience, the air samples were rarely contaminated with the ship's exhaust, when the ship sails at approximately 20 knot because the ship's exhaust is located at stern side. When the air samples were contaminated with the exhaust gas, we judged it by the CO_2_ and O_3_ measurements (CO_2_ increase and O_3_ decrease), and then rejected the data before the analysis.

For instrument calibration, we prepared a set of three natural or purified air-balanced standard gases with CO_2_ and CH_4_ (ca. 380, 400, and 420 ppm for CO_2_, and 1800, 2000, and 2200 ppb for CH_4_) in our laboratory to prevent pressure-broadening effects due to the different air compositions of the samples and standard gases. The standard gases were introduced into the WS-CRDS instrument daily in series, 10 min for each gas. The mole fractions of CO_2_ and CH_4_ in the standard gases were calibrated against NIES standard gas scales (NIES 09 for CO_2_, NIES 96 for CH_4_), which are traceable to World Meteorological Organization standard gas scales. The analytical precision for 1-min measurements by the WS-CRDS instruments of CO_2_ and CH_4_ were typically 0.05 ppm and 0.5 ppb (1 sigma), respectively. In this study, we used only the 1-min temporal mean CH_4_ and CO_2_ data from the continuous measurements because of the coarse resolution of the CO data, available only as 1-hour means from the gas filter correlation measurements, and because the O_3_ data provide little information about CH_4_ emission sources.

## Author Contributions

H.N. and H.T. took the main role in the study design and the data analyses. Y.T. actively participated in the analyses and discussion, H.M. and Y.N. took the leading role in the operation of the monitoring project, T.M. took the key role in the assurance of data quality. All authors participated in the discussion of results and H.N., H.T. and Y.T. wrote the manuscript.

## Figures and Tables

**Table 1:** Estimated CH_4_ emission rates for the observed CH_4_ peaks

Area	Peak No.	*u* (m s^−1^)	α (deg.)	Z_MBL_ (m)	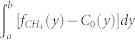 (ppm m × 10^4^)	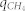 (g s^−1^)
Malay	M1	2.4	38.0	565	1.4	38.1 (9.1–89.3)
	M3	2.8	2.1	252	10.0	180.8 (43.3–423.8)
	M4	5.0	23.4	430	2.6	133.0 (31.9–311.8)
	M5	5.1	12.2	556	0.23	16.8 (4.0–39.4)
	M6	5.8	25.4	505	1.6	110.7 (26.5–259.5)
	M7	2.1	46.5	574	20.2	426.7 (102.2–1000.0)
	M8	2.2	7.5	607	0.11	3.9 (0.9–9.2)
	M9	2.6	8.9	677	1.9	87.7 (21.0–205.5)
Borneo	B1	3.6	82.1	517	7.0	46.0 (11.0–107.9)
	B6	3.4	9.9	469	0.43	17.5 (4.2–41.1)
	B11	0.8	11.7	651	0.31	4.0 (1.0–9.3)
	B12	3.9	78.9	597	0.16	1.8 (0.4–4.3)
	B13	3.9	78.0	588	1.6	20.2 (4.9–47.5)
	B14	3.0	78.9	674	1.2	11.8 (2.8–27.6)

The value of 1.2 kg m^−3^ is used for the molar density of air to calculate the CH_4_ emission rates. Figures in parentheses are the uncertainty ranges.
